# Interpreting sperm DNA damage in a diverse range of mammalian sperm by means of the two-tailed comet assay

**DOI:** 10.3389/fgene.2014.00404

**Published:** 2014-11-27

**Authors:** Elva I. Cortés-Gutiérrez, Carmen López-Fernández, José Luis Fernández, Martha I. Dávila-Rodríguez, Stephen D. Johnston, Jaime Gosálvez

**Affiliations:** ^1^Department of Genetics, Centro de Investigación Biomédica del Noreste, Instituto Mexicano del Seguro SocialMonterrey, México; ^2^Unit of Genetics, Department of Biology, Universidad Autónoma de MadridMadrid, Spain; ^3^Section of Genetics-INIBIC, Hospital Teresa Herrera, Complejo Hospitalario Universitario A CoruñaA Coruña, Spain; ^4^School of Agriculture and Food Science, The University of QueenslandGatton, QLD, Australia

**Keywords:** Sperm DNA damage, fertility, male factor, mammalian reproduction, eutheria, metatheria, prototheria

## Abstract

**Key Concepts**

- The two-dimensional Two-Tailed Comet assay (TT-comet) protocol is a valuable technique to differentiate between single-stranded (SSBs) and double-stranded DNA breaks (DSBs) on the same sperm cell.
- Protein lysis inherent with the TT-comet protocol accounts for differences in sperm protamine composition at a species-specific level to produce reliable visualization of sperm DNA damage.
- Alkaline treatment may break the sugar–phosphate backbone in abasic sites or at sites with deoxyribose damage, transforming these lesions into DNA breaks that are also converted into ssDNA. These lesions are known as Alkali Labile Sites “ALSs.”
- DBD–FISH permits the *in situ* visualization of DNA breaks, abasic sites or alkaline-sensitive DNA regions.
- The alkaline comet single assay reveals that all mammalian species display constitutive ALS related with the requirement of the sperm to undergo transient changes in DNA structure linked with chromatin packing.
- Sperm DNA damage is associated with fertilization failure, impaired pre-and post- embryo implantation and poor pregnancy outcome.
- The TT is a valuable tool for identifying SSBs or DSBs in sperm cells with DNA fragmentation and can be therefore used for the purposes of fertility assessment.

Sperm DNA damage is associated with fertilization failure, impaired pre-and post- embryo implantation and poor pregnancy outcome. A series of methodologies to assess DNA damage in spermatozoa have been developed but most are unable to differentiate between single-stranded DNA breaks (SSBs) and double-stranded DNA breaks (DSBs) on the same sperm cell. The two-dimensional Two-Tailed Comet assay (TT-comet) protocol highlighted in this review overcomes this limitation and emphasizes the importance in accounting for the difference in sperm protamine composition at a species-specific level for the appropriate preparation of the assay. The TT-comet is a modification of the original comet assay that uses a two dimensional electrophoresis to allow for the simultaneous evaluation of DSBs and SSBs in mammalian spermatozoa. Here we have compiled a retrospective overview of how the TT-comet assay has been used to investigate the structure and function of sperm DNA across a diverse range of mammalian species (eutheria, metatheria, and prototheria). When conducted as part of the TT-comet assay, we illustrate (a) how the alkaline comet single assay has been used to help understand the constitutive and transient changes in DNA structure associated with chromatin packing, (b) the capacity of the TT-comet to differentiate between the presence of SSBs and DSBs (c) and the possible implications of SSBs or DSBs for the assessment of infertility.

## Introduction

Different methodologies exist to detect DNA breaks in somatic and sperm cells. Some of these techniques are based on the propensity of the DNA molecule to form single stranded DNA stretches in the presence of stressing environments such as alkaline or acid solutions. Alkaline sucrose gradient sedimentation, alkaline elution or alkaline DNA precipitation, are biochemical techniques based on alkaline DNA unwinding that have been used to assess the presence of single-strand DNA breaks (SSBs). Variants also exist where the DNA molecule is processed under non-denaturing buffered conditions; under these pH conditions, detection of double-strand DNA breaks (DSBs) is feasible, especially when the DNA molecule is strongly de-proteinized prior to electrophoresis (Ahnstrom, 1988; Olivie, 2006; Olive and Banáth, 2007). DSBs seem to be more relevant to the production of chromosome aberrations and can arise as a consequence of insufficient or inefficient DNA repair activity to restitute the original linear chromosomal DNA continuity; these chromosomal rearrangements or deletions may result in stoppage or delay of the cell cycle, and cell death (Marchetti et al., 2007).

Intercellular heterogeneity in DNA damage production or repair can be assessed *in situ* using morphological procedures such as the single-cell electrophoresis assay commonly known as the comet assay (McKelvey-Martin et al., 1993; Collins, 2004). The comet assay is a straightforward method for assessing DNA strand breaks in eukaryotic cells and the methodology is relatively simple. Basically, live cells can be embedded into a microgel on a microscope slide, lysed with a controlled high salt and detergent solution to form nucleoids which are visible under fluorescence microscopy and which form a comet image after migration of DNA fragments associated with electrophoresis. The intensity of migrated DNA at the comet tail, relative to the head, is a directly linearly related to the quantity of DNA breaks originally present in the DNA molecule (Collins, 2004). The original comet assay can be considered as modification of the “halo” assay as conceived by Cook et al. (1976); the connection between the concept of “halo” and the “comet” emerged from Ostling and Johanson (1984) some 8 years later. The first version of the comet assay was performed under neutral conditions but using relatively low strength protein removal; this is interesting, because under these conditions, the morphology of the comet has been found to be highly dependent on the capacity of the protein depletory agents to induce chromatin relaxation of a supercoiled DNA molecule. A new modification of the original neutral comet assay, as conceived by Ostling and Johanson (1984), was developed by Singh et al. (1988), but in this case, electrophoresis was performed under an alkaline-DNA denaturant environment. The rationale of this methodology was to mobilize single stranded DNA molecules unwound from the end of the breaks. Discrepancies exist in the literature as to what is the “real” information derived from the different assays in terms of DNA break production (SSBs or DSBs) because the scenarios for which the techniques have been used (see examples in Collins, 2004) are as different as the chromatin organization of the cells subjected to analysis. It is not surprising that the behavior of somatic cell and gametic chromatin to equivalent treatments varies so dramatically when you consider the different levels of tissue dependent heterochromatinization, the highly histonized nature of somatic chromatin and the genetic inactivity and histone replacement by protamines during spermatogenesis.

## The two-tailed (TT) comet assay—the importance of species-specific protein lysis

The possibility exists of combining non-denaturing and denaturant conditions to the same sperm nucleoid. In this case, the species-specific de-proteinized sperm is first subjected to an electrophoretic field under non-denaturing conditions to mobilize isolated free discrete DNA fragments produced from DSBs; this is then followed by a second electrophoresis running perpendicular to first one but under alkaline unwinding conditions to produce DNA denaturation exposing SSBs on the same linear DNA chain or DNA fragments flanked by DSBs. This procedure results in a two dimensional comet tail emerging from the core where two types of original DNA affected molecule can be simultaneously discriminated. The two-dimensional perpendicular tail comet assay (TT-comet) is an excellent methodological approach to distinguish between single and double strand DNA damage within the same cell. In this review, we present TT-comet assay data that our group has published for the three sub-classes of mammals, the prototheria (echidna), metatheria (koala and kangaroo) and eutheria (Enciso et al., 2009; Johnston et al., 2009; Enciso et al., 2011a,b; Portas et al., 2009; Zee et al., 2009; Gosálvez et al., 2014).

The difference in sperm chromatin structure is particularly fascinating as each group has a different protamine amino-acidic composition (Table 1) (Vilfan et al., 2004) so that lysing solutions used in the preparation of the TT-comet in order to produce a controlled protein depletion need to be targeted and species-specific to make the analyses comparable. There are two major amino acid residues in protamines that appear to be important for understanding DNA and protein assembly associated with sperm chromatin compactness; these include (a) the presence of cysteine residues that allow the formation of intra- and inter-disulphide bonds and (b) the existence of arginine residues that permit more intense positive or negative charged protamines to interact with the sperm DNA. Species differences in protamine sequences are illustrated in Table 1. The relative composition and location of these particular residues in the sperm DNA of the different mammalian taxa combined with their respective relationship to the interspecific heterogeneity of protamine 1/protamine 2 ratio, and the arrested substitution of protamine 1 by protamine 2 (e.g., boar and bull), highlights the uniqueness of sperm DNA molecule when compared to the rest of the soma (Biegeleisen, 2006; Balhorn, 2007; Gosálvez et al., 2011). This phenomenon also makes the comparative investigation of sperm DNA from species other than human or domestic animals experimental model for understanding DNA packaging and fragmentation.

**Table 1:** **Multiple amino-acid sequence alignment in different mammalian species to show species-specific differences in cysteine residues and arginine content in Protamine 1**.

**Species**	**English name**	**Amino acid reference and alignment**				**NR**	**C**	**A**
*Homo sapiens*	Human	MARYRC CRSQSRSRYY RQRQRS	RRRR	RRSCQTRRRAMRCCR	PRYRPRCRRH	51	6/2	24
*Pan troglodytes*	Chimpanzee	MARYRC CRSQSRSRCY RQRQRS	RRRK	RQSCQTQRRAMRCCR	RRSRMRRRRH	51	6/2	23
*Orictolagus cuniculus*	Rabbit	MVRYRC CRSQSRSRCR RRRRR	CRRR	RRRCCQRRRVRKCCR	RTYTLRCRRY	50	9/3	26
*Felis catus*	Cat	MARYRC CRSHSRSRCR RRRRR	CRRR	RRRCC RRPRKRVCSR	RYRVGRCRRR	50	8/2	28
*Ursus arctos*	Bear	MARYRC CRSHSRSRCR RRRRR	CRRR	RRRCCRRRRRRVCCR	RYTVVRCRRR	50	9/3	29
*Camelus bactrianus*	Camel	MARYRC CRSHSRSRCR PRRRR	CRRR	RRRCCRRRRRRVCCR	RYTIIRCRRR	50	9/3	28
*Sus scrofa*	Boar	MARYRC CRSHSRSRCR PRRRR	CRRR	RRRCCPRRRRAVCCR	RYTVIRCRRC	50	10/3	25
*Orcinus orca*	Killer Whale	MARNR CRSPSQSRCR RPRRR	CRRR	IRCC RRQRRVCCR	RYTTTRCARQ	47	8/2	21
*Equus asinus*	Donkey	MARYRC CRSQSQSRCR RRRRRR	CRRR	RRRC VRRRRVCCR	RYTVLRCRRRR	50	8/1	28
*Equus caballus*	Stallion	MARYRC CRSQSQSRCR RRRRRR	CRRR	RRRS VRQRRVCCR	RYTVLRCRRRR	50	7/2	27
*Bos taurus*	Bull	MARYRC CLTHSGSRCR RRRRRR	CRRR	RRRSGRRRRRRVCCR	RYTVIRCTRQ	51	7/2	26
*Alces alces*	Moose	MARYRC CLTHSRSRCR RRRRRR	CRRR	RRRFGRRRRRRVSCR	RYTVIRCTR	50	6/2	27
*Capra hircus*	Goat	MARYRC CLTHSRSRCR RRRRRR	CRRR	RRRFGRRRRRRVCCR	RYTVVRCTRQ	51	7/2	27
*Gazella dorcas*	Gazelle	MARYRC CLTHSRSRCR RRRRRR	CHRR	RRRFGRRRRRRVCCR	RYTVVRCTRQ	51	7/2	26
*Ovis aries*	Ram	MARYRC CLTHSRSRCR RRRRRR	CRRR	RRRFGRRRRRRVCCR	RYTVVRCTRQ	51	7/2	27
*Ratus norvergicus*	Rat	MARYRC CRSKSRSRCR RRRRR	CRRR	RRRCCRRRR-RRCCRR	RRSYTFRCKRY	51	9/3	29
*Mus musculus*	Mouse	MARYRC CRSKSRSRCR RRRRR	CRRR	RRRCCRRRR RRCCRR	RSYTIRCKKY	51	9/3	28
*Loxodonta africana*	Elephant	MARYRC CRSRSRSRCRSRRRRRS	HRRR	RRCARRRRRTRRGCR	RRYSLRRRRY	52	5/1	31
*Phascolarctos cinereus*	Koala	MARYRH SRSRSRSRYQRRRRRRSRYRSQRRRYRRRRGSRRRRRRGRRRG YRRRYSRRRRY				60	0	37
*Planigale maculate sinualis*	Planigale^*^	MARCRRHSRSRSRSRNQCQRRRR RRYNRRR TYRRSRRHSRRRRGRRRGCSRRRYSRRGRRRY				62	3	35
*Planigale maculate muculata*	Planigale^*^					63	0	37
*Sminthopsis crassicaudata*	Dunnart	MARYRRHSRSRSRSRYRRRRRRRS RHHNRRR YRRSRRHSRRRRGRR RGYSRRRYSRRGRRRY						
*Didelphis virginiana*	Opossum	MARYRRRSRSRSRSRYG RRRR RSRSRR RRSRRR RRRRGRRGRGYHRRSPHRRRRRRRR				58	0	38
*Tachyglossus aculeatus*	Echidna	MARFRP SRSRSRSLYRRRRRSRR QRSRRGGRQTGPRKITRRGRGRGKSRRRRGRR SMRSSRRRRRRRRN				69	0	36

NR, Number of residues; C, Cysteine; A, Arginine;

* *Subspecies of the Common Planigale*.

Large structural differences exist between somatic cells and spermatozoa. For example, the replacement of histones by protamines in the sperm cell facilitate; (a) the efficient chromatin packaging to provide additional protection of the DNA during the long journey in the female reproductive tract and (b) the production of an ATP driven flagellum for autonomous displacement capacity. In the majority of mammalian species, especially in eutherian mammals, cysteine residues are present in the protamines to create a more condense and well-packed chromatin fiber, so that when the comet assay is performed under either neutral or alkaline conditions, there is a requirement to first pretreat the chromatin to loosen this protective protein by means of a reducing agent (dithiothreitol or beta-mercaptoethanol). This treatment specifically reduces the covalent disulphide (SS) bridges present at both the intra-protamine and inter-protamine molecular level (Bedford and Calvin, 1974a; Yanagimachi, 1994; Vilfan et al., 2004), so that any putative free DNA fragments can be mobilized under an electrophoretic field. An understanding of the inherent peculiarities of sperm DNA structure between the different species of mammals is of fundamental importance when establishing sperm comet assays in novel taxa, so that the technique needs to be appropriately validated for each species in order to account for differences in chromatin structure.

## Sperm DNA comet assay under non-denaturing neutral conditions

**Figure 1:**
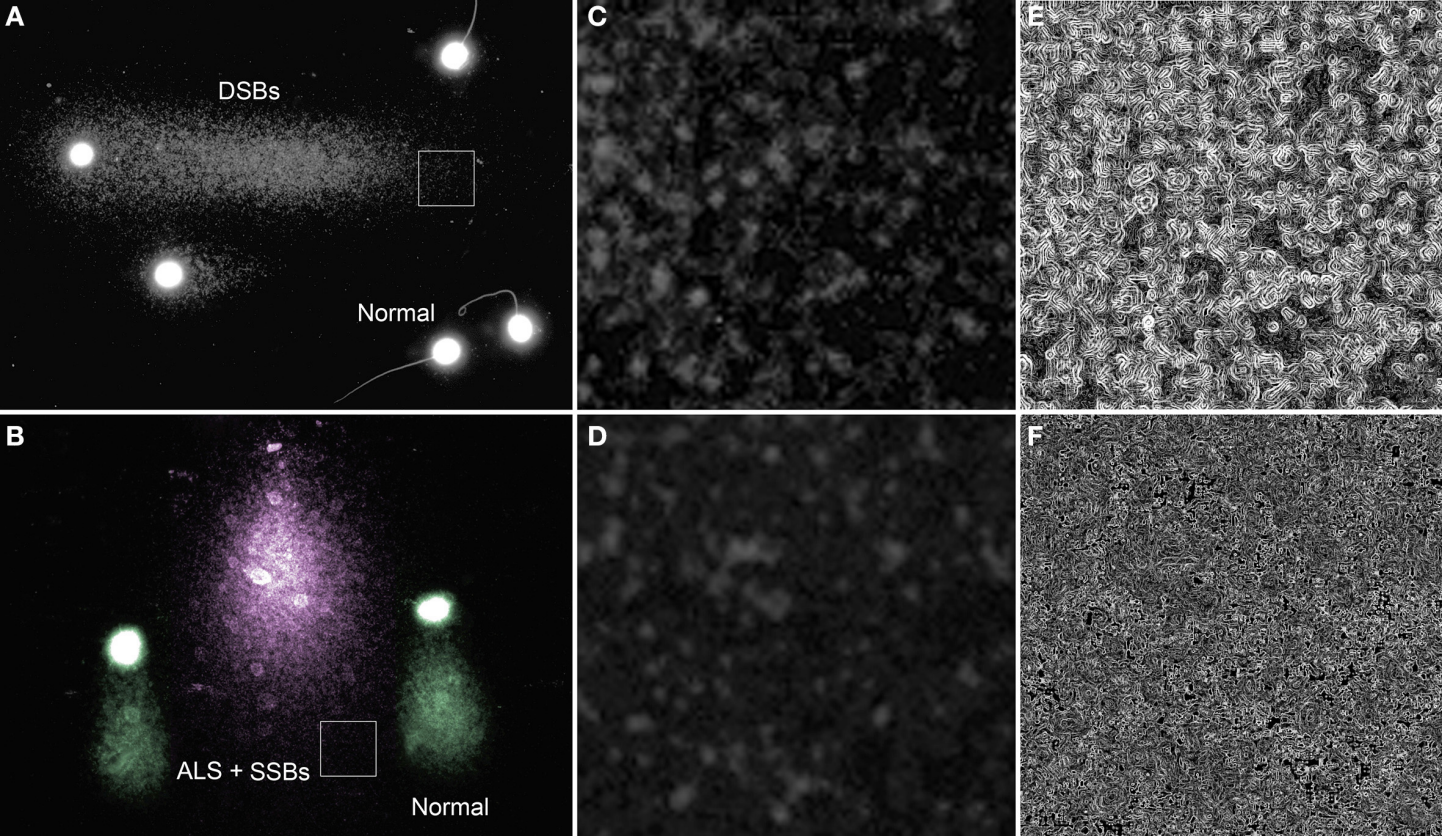
**Single neutral and alkaline comets. (A)** Neutral comet from rhinoceros *(Rhinoceros sondaicus)* sperm showing a tail of mobilized DNA fragments (right direction of the image) as a consequence of double strand DNA breaks (DSBs) at the origin of the sperm head. Sperm without comet tail (labeled as normal for this condition) do not contain detectable levels of DSBs, but display a small halo of compact chromatin. **(B)** Alkaline comet of rhinoceros sperm (pink comet) showing a tail of mobilized DNA fragments which are interpreted as single strand DNA stretches derived from short DSBs susceptible to be denatured, single breaks (SSBs) and alkali labile associated with structural comets (pseudo-colored green). Magnified regions (box) within the neutral **(C)** and an alkaline comet tail **(D)** are provided to visualize the difference in the chromatin structure along with filtered images to enhance differences in chromatin texture (**E**—neutral and **F**—alkaline).

When lysed spermatozoa with no DNA fragmentation are subjected to an electrophoresis under non-denaturing conditions, no substantial comet tails are formed (sperm labeled as normal in Figure 1A). In contrast, damaged sperm DNA show extensive migration of DNA fragments from the original sperm core (Figure 1A) and these migrating DNA fragments are most likely to be associated with DSBs which were present at the origin; however, one needs to be cautious about this interpretation as these DNA fragments are also likely to contain “internal” single strand breaks that cannot be differentiated. Similar comets can be produced after incubation with classic double strand DNA cutters such as restriction endonucleases. Alu I, for example, is an enzyme that is able to selectively identify and cleave CGTT sites on fixed chromatin (Mezzanotte et al., 1983; Miller et al., 1983) producing substantial DNA release. Restriction endonuclease gives rise to specific DSBs and the extensive production of comets, which is the direct consequence of DNA cleavage produced by enzymatic treatment (Brooks, 1987).

## Sperm DNA comet under alkaline denaturing conditions

### Determination of structural comets and alkali labile sites in spermatozoa

**Figure 2:**
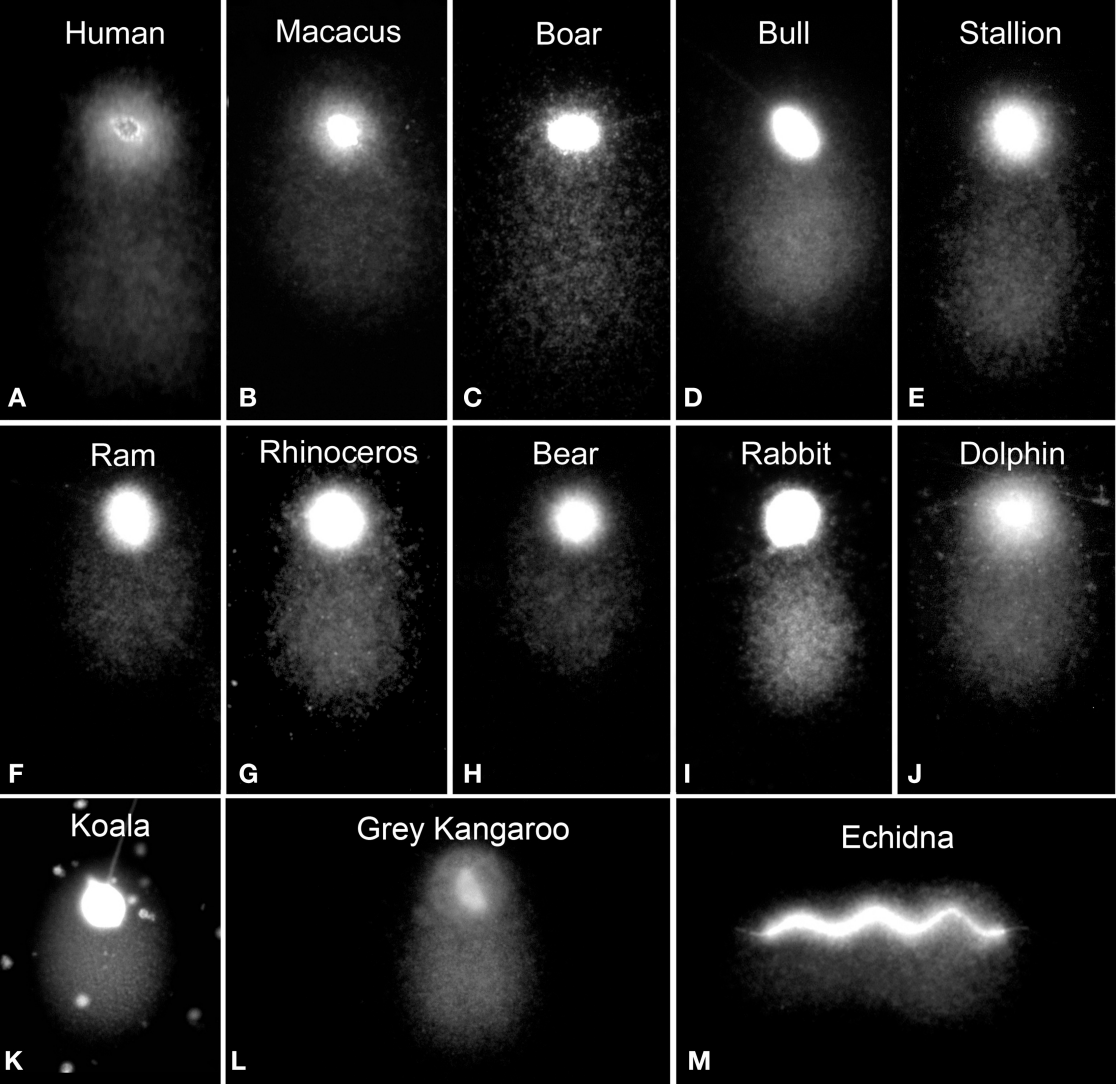
**Structural alkaline comets in different mammalian sperm **(A–J)** eutherian species, **(K,L)** metatherian species and **(M)** a prototherian species**.

DNA breaks are starting points for alkaline DNA unwinding due to the disruption of hydrogen bonds among purines and pyrimidines. Moreover, mutagens may induce DNA base loss and deoxyribose lesions that may be transformed into SSBs by alkaline conditions, being designated as alkali labile sites (ALS). Remarkably, when the spermatozoa of all mammalian species so far analyzed are subjected to denaturant alkaline conditions and electrophoresed, they exhibit a prominent comet tail (Singh and Stephens, 1998; Fernández et al., 2000; Cortés-Gutiérrez et al., 2009) (Figure 2). These structural comets are present in the sperm cells of all three different sub-classes of mammals (Figure 2). Differences in the length of the tails are observed when different eutherian species are compared; for example, compare human, macacus (*Macacus rehesus*), boar, bull, stallion, ram, rhinoceros, bear, rabbit, dolphin (*Delphinus delphis*) (Figures 2A–J respectively); these comer tails appear to be comparatively shorter in the spermatozoa of metharian species subjected to the same experimental conditions; koala and gray kangaroo (*Macropus fuliginosus*) (Figures 2 K,L, respectively) and in the case of echidna (prototheria), which possesses a unique elongated filiform sperm nucleus, with the comet being observed along the length of the sperm head (Figure 2M). Mammalian sperm chromatin appears especially susceptible to alkaline “breakage” and/or denaturation, representing the presence of a high density of SSBs or ALS. Comets after DNA denaturation show a more diffuse chromatin and the visualized DNA fragments at the end of the tail are not as defined as those produced after neutral comets [compare Figures 1C,E (neutral) with Figures 1D,F (alkaline)]. The presence of a comet tail associated with SSBs or ALSs is not related to any harmful DNA damage but is a consequence or a feature inherent to the sperm chromatin structure; we refer to these comets as “structural” or “constitutive” comets.

**Figure 3:**
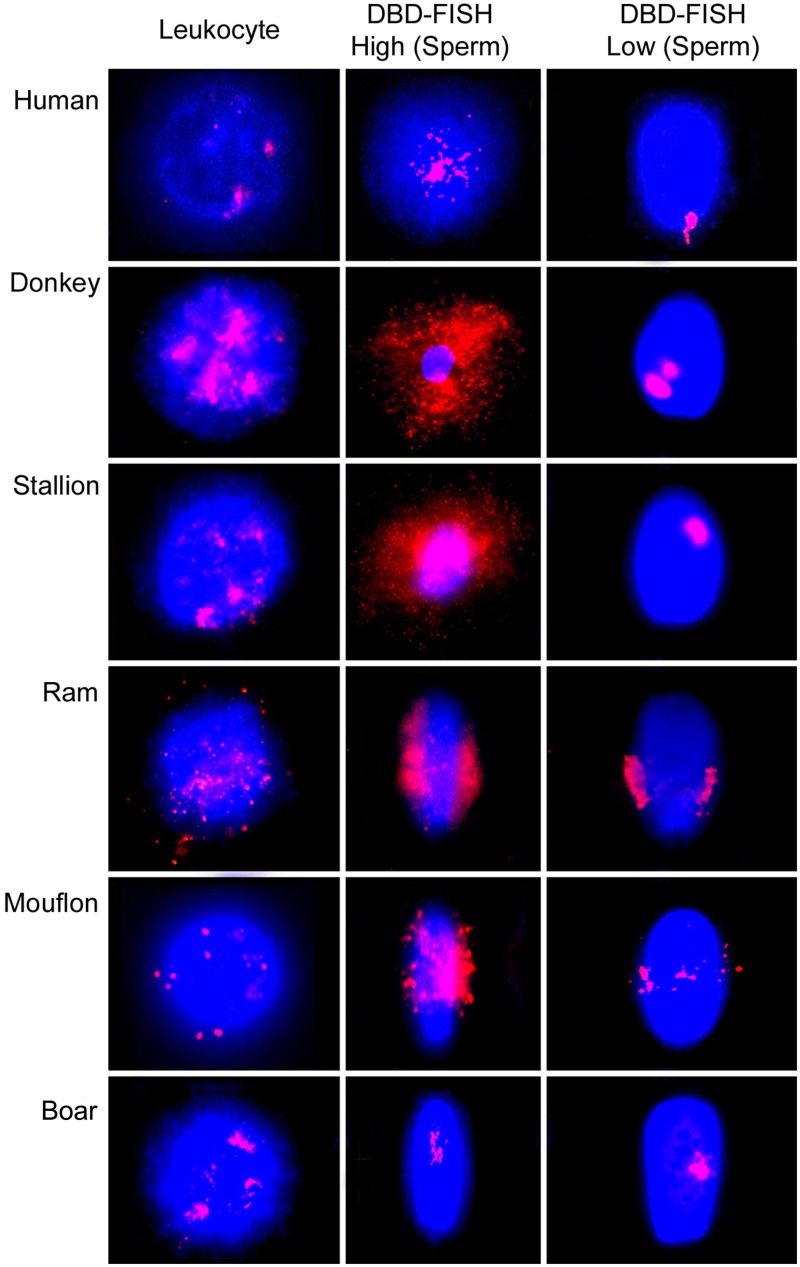
**Visualization of the alkali labile sites (ALS) using DNA Breakage Detection-Fluorescence *In Situ* Hybridization (DBD-FISH) as a structural feature of the spermatozoa and peripheral blood leukocytes in different mammal species**. High alkali denaturation conditions (DBD-FISH High) show most of the ALS present al the spermatozoa. Mild alkaline conditions (DBD-FISH Low) revealed the localization of regions most sensitive to alkaline denaturation.

Alkali labile sites can also be detected using the DNA Breakage Detection-Fluorescence *In Situ* Hybridization (DBD-FISH) procedure (Fernández et al., 2000; Fernández and Gosálvez, 2002). Using this procedure, cells embedded within an inert agarose matrix on a slide are lysed and the resultant nucleoids exposed to a controlled alkaline denaturation step. Under these conditions, putative DNA breaks are transformed into restricted single-stranded DNA motifs, initiated from the ends of the DNA breaks that may be detected by hybridization using either whole genome or specific fluorescent DNA probes. The specific DNA probe selects the chromatin area to be analyzed. As DNA breaks increase within a specific target, more single-stranded DNA is generated and more DNA probe hybridizes, producing increasing levels of fluorescence (Fernández et al., 2000; Fernández and Gosálvez, 2002) (Figure 3). It is noteworthy that when a whole-genome DNA probe is hybridized to somatic cells, the background DBD-FISH signal is not homogeneous and certain chromatin regions are selectively and strongly labeled; this is especially evident when high levels of alkali denaturation are used on the native sperm chromatin (Figure 3, DBD-FISH High). It is of interest to highlight that the DNA sequences related with constitutive ALS mostly correspond with certain specific highly repetitive DNA sequences (Fernández et al., 2001; Rivero et al., 2001, 2004). In human leukocytes, the more intense background DBD-FISH areas within the genome correspond to DNA domains containing 5-bp satellite DNA sequences (Fernández and Gosálvez, 2002). In mouse splenocytes, the background labeled areas correspond with highly repetitive major DNA satellite sequences located in pericentromeric regions (Rivero et al., 2001), and in Chinese hamster cells, they match to pericentromeric interstitial telomeric-like DNA sequence blocks (Rivero et al., 2004). As indicated, all these native highly alkali-sensitive regions correspond to strongly compacted chromatin domains present in somatic nuclei.

It is also possible to control assay conditions to produce DNA denaturation using very mild alkaline to produce very restrictive single strand DNA stretches to be revealed later by DBD-FISH (See Figure 3 DBD-FISH-low). Using this approach, it can be demonstrated that the most sensitive genome regions to alkaline denaturation are not randomly localized in the sperm nucleus. Usually there is one or two discrete genome domains consistently localized in each species, but these regional locations differ amongst species. For example, in human spermatozoa, the most alkali sensitive region appears located at the proximal end of the spermatozoon, adjacent to the implantation fossa (Cortés-Gutiérrez et al., 2014a). In other species such as the ram, ALSs are located along the equatorial region of the sperm head and consist of two opposing clusters of hybridization. In the case of the stallion, ALS show a tendency to cluster, but the closely related donkey presents two discrete clusters of hybridized signal. Both signals tended to be localized at the equatorial-distal regions of the sperm. Boar spermatozoa present a discrete signal localized in the central region of sperm (Cortés-Gutiérrez et al., 2009).

The differences in the quantity of ALSs between somatic cells and spermatozoa can also be evidenced using DBD-FISH (Fernández et al., 2000; Fernández and Gosálvez, 2002; Cortés-Gutiérrez et al., 2009) (Figure 3). Using this technique, the hybridization signal produced with a whole-genome DNA probe is 12.7 times more intense in spermatozoa than the signal obtained in peripheral blood leucocytes (Muriel et al., 2004). In mouflon (*Ovis musimon*), the density of ALS in sperm is eight times higher than that of the somatic cells. In sheep, both leucocytes and sperm cells exhibited a large quantity of ALS, being four times more abundant in sperm (Cortés-Gutiérrez et al., 2008). In donkey and stallion, the relative abundance of ALSs was also four times higher in spermatozoa than in somatic cells. ALSs in the sperm of donkey was 1.3 times greater than in stallion and the length of the comet tail obtained in donkey sperm was 1.6 times longer than that observed in horse and the differences were significant (*P* < 0.05) (Cortés-Gutiérrez et al., 2014b). Boar spermatozoa are unique in this respect as ALSs are 12 times higher in their leukocytes compared to spermatozoa (Cortés-Gutiérrez et al., 2008). Interestingly, only the satellite DNA sequences integrated at the pericentromeric heterocromatin of all metacentric chromosomes of the karyotype were contributing to produce ALSs in the boar (Cortés-Gutiérrez et al., 2008). Later it was found that the low quantity of ALS detected after DBD-FISH in the boar was in fact an artifact linked to the limited ability of the alkaline denaturation and protein lysis used for the DBD-FISH procedure and the extremely strong compacted chromatin structure present in the boar sperm cell, and which is dependent on only protamine 1 and which presents with 5 cysteine residues per protamine molecule (Gosálvez et al., 2011).

We propose that structural sperm comet tails can be interpreted as a consequence of the peculiar massive presence of constitutive ALSs in natural chromatin and is likely to be a manifestation of a physical and transient circumstance linked to the specific need for efficient chromatin packing (Allen et al., 1997). These regions also seem to be especially susceptible to *in situ* enzymatic digestion by “mung bean nuclease” so could correspond to stretches of partially denatured single-stranded DNA, which could act as starting points of DNA denaturation by alkaline conditions (Bedford and Calvin, 1974b; Fernández et al., 2000; Cortés-Gutiérrez et al., 2014a). While it has not been fully demonstrated whether these regions correspond to abasic (apurinic or apyrimidinic) sites that can be converted into DNA breaks by the alkali, this possibility certainly exists; in fact, spermatozoa are quite recombinogenic in the presence of exogenous DNA (Sakkas et al., 2002; Fernández-González et al., 2008).

**Figure 4:**
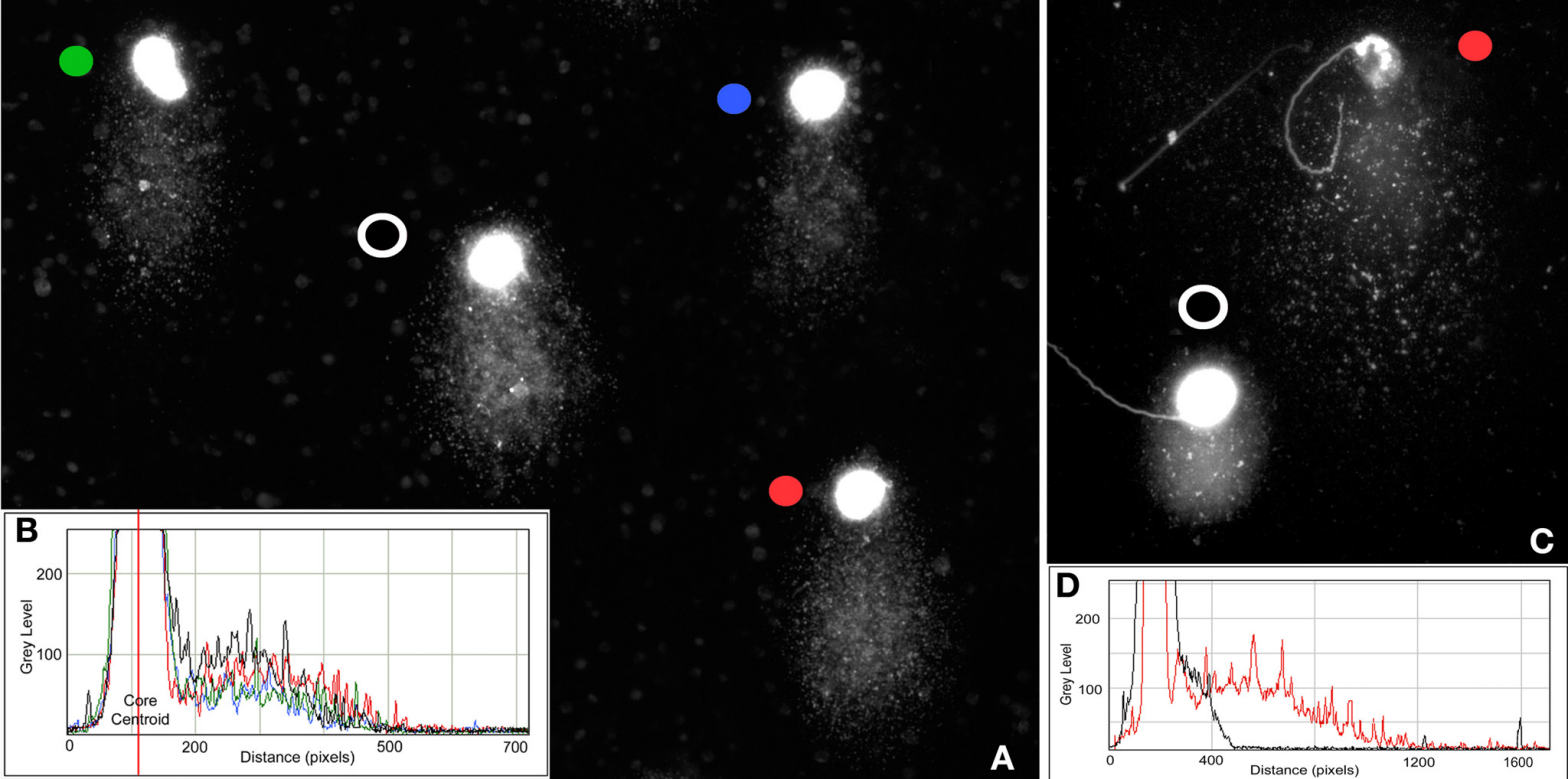
**(A)** Stallion structural comets showing differences in the density of DNA but similar tail length; **(B)** Density profile of horse comets showing differences in fluorescence intensity—different colored circles correspond to the colored graphic profiles of individual spermatozoa; **(C)** Structural (black dot) and damaged (red dot) koala spermatozoa showing differences in the amount of DNA and DNA migration distance; **(D)** Density profile of koala comets showing differences in fluorescence intensity and length of comet tail.

Differences also exist in the length of the structural comets observed among different species with shortest found in the bear and koala (Figure 2). This phenomenon has not been studied in detail, although the most parsimonious hypothesis to assume would be that the size of the structural comet is related to the differential susceptibility of the chromatin in each species to an equivalent treatment to produce DNA denaturation; this phenomenon deserves more thorough investigation. Interestingly, within each species, structural comets do not show large differences in comet tail length (Figure 4A) but it is possible to detect differences in their respective fluorescence intensity as illustrated in the accompanying profiles (Figures 4A,B).

### Differentiation of induced mutagenic SSBs from constitutive ALS

Under alkaline conditions, structural comet tails associated with ALS can be differentiated from “real” DNA damage by observing the density and length of the comet tail. Sperm nuclei with “real” denatured DNA derived from DSBs, SSBs and ALSs have significantly longer comet tails (pink comet in Figure 1B) than those that are merely structural comets (green comets in Figure 1B). Figures 4A,B show the difference in the DNA density on sperm comets in stallion conducted under alkaline conditions, although the migration distance from the core is quite similar in all the cases. In Figures 4C,D, we show the differences in both DNA density in the comet and comet tail length as visualized in koalas. In this case, the difference in the DNA migration between affected and non-affected sperm is prominent because the structural comet in this species is not as large as that observed in other eutherian species. When the sperm is incubated with agents that primarily induce SSBs such as hydrogen peroxide (Yamamoto, 1969) or sodium nitroprusside (a nitric oxide donor) (Lin et al., 2000; Ichikawaa et al., 2008), highly enlarged comet tails emerge from the core following denaturing conditions (Lin et al., 2000). The length of the tail as well as the intensity profile of staining of the DNA migrated from the core is related to the amount of induced damage.

## Two tail comet conducted under sequential neutral and alkaline conditions

**Figure 5:**
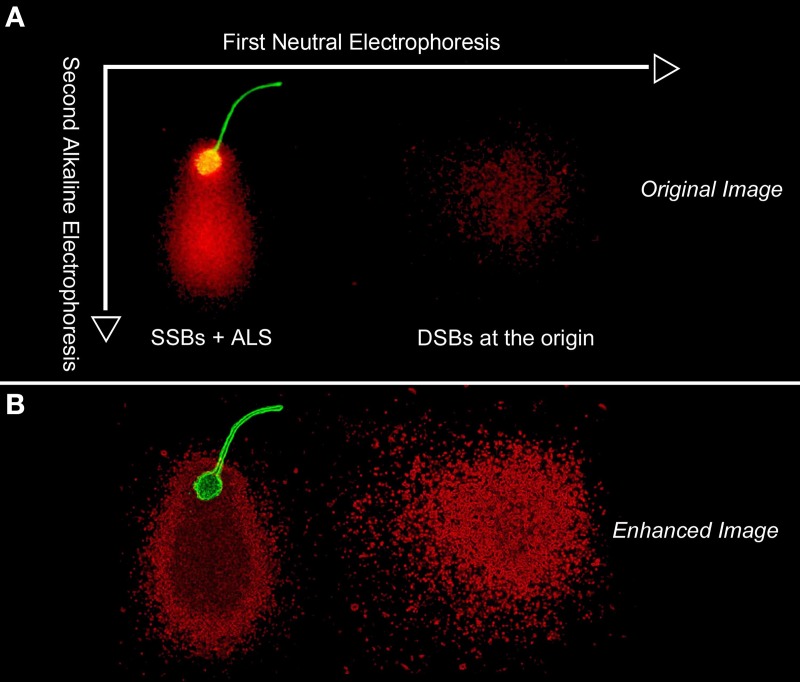
**Original (A) and digitally enhanced image (B) to show whole DNA fragment distribution DNA map following a two tailed comet assay in a human sperm cell**. The first neutral electrophoresis results in a horizontal migration along the X axis of DNA fragments formed as a consequence of DSBs. After turning the microgel 90°, the second electrophoresis results in the migration of DNA along the vertical Y axis and is conducted under alkaline conditions; the alkaline comet assay reveals both structural Alkali Labile Sites (ALSs) and “true” SSBs that have elongated comet tails. The green fluorescence is associated with proteinaceous remnants of the sperm head and flagellum.

Figure 5 shows a TT-comet of a human spermatozoa as visualized under fluorescence microscopy (original image -5A- and electronically filtered -5B-) and the putative distribution of the DNA breakage present in the original spermatozoon. To produce a TT comet, deproteinized sperm are initially subjected to a neutral electrophoresis that results in the mobilization of free DNA-chromatin fragments associated with DSBs along the X-axis. While the DNA domain on the X axis represents DSBs at the origin, the tail may also contain SSBs that could potentially be denatured when exposed to the later alkaline conditions and run in a second electrophoresis at 90° to the first one; under these altered conditions, the DNA fragments would then migrate along the Y axis; this phenomenon is well illustrated in the enhanced image (Figure 5B). The comet tail emerging from the sperm nuclear core along the Y-axis contains single stranded DNA stretches, which were produced after denaturing single and double strand breaks existing at the origin that were not displaced during the neutral electrophoresis. Initially, they could be long double-stranded DNA fibers containing DNA nicks at different positions in both strands, but they were too large to be mobilized during the first non-denaturing electrophoresis; however now under alkaline conditions, they can be readily mobilized from the core after DNA denaturation, migrating perpendicularly to the first electrophoretic run. DNA molecules forming the comet tail during the first neutral electrophoresis on the X axis are similarly denatured and displaced along the Y axis; they represent a cloud of single strand DNA stretches emerging from a primary cloud of non-denatured DNA formed from double strand breaks at the origin.

**Figure 6:**
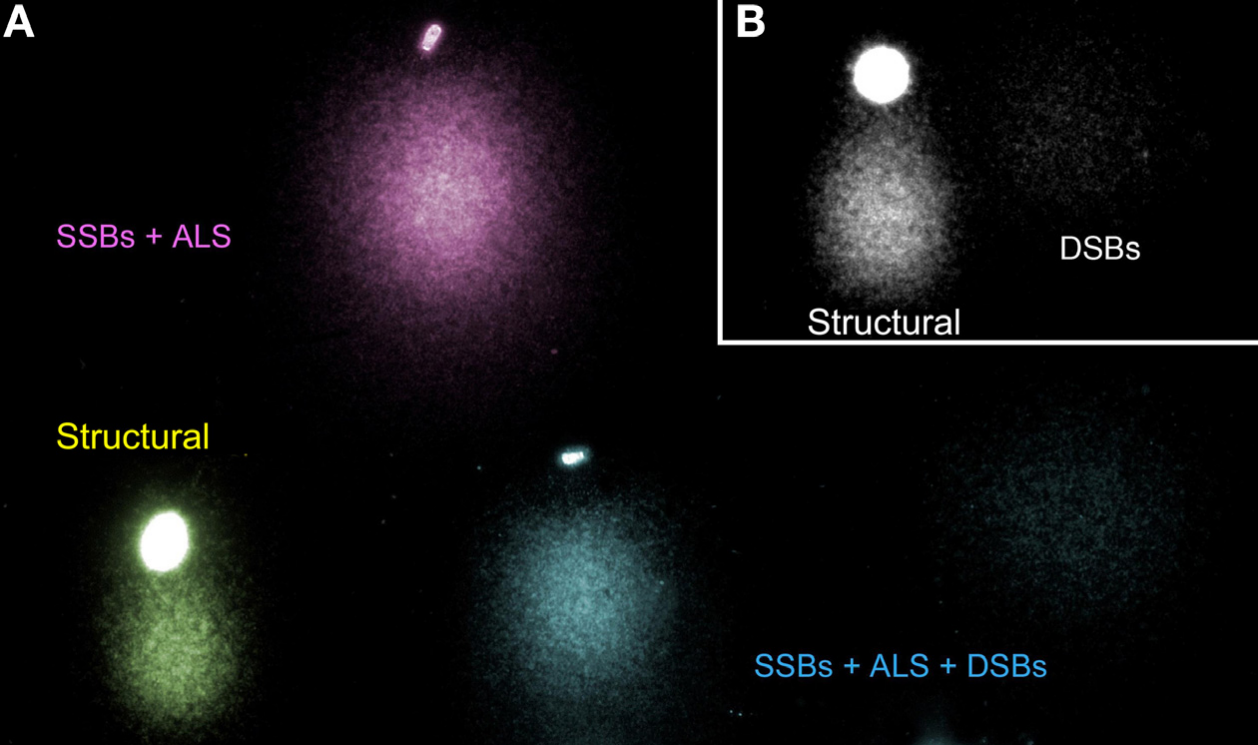
**Pseudo-colored images of sperm DNA following a TT comet assay and illustrating different comet morphology associated with different types of DNA damage. (A)** Type 1 (yellow)—structural comet; Type 2 (pink)—SSBs plus ALSs; Type 3 (blue) DDBs, SSBs plus ALS within the same TT comet and **(B)** Type 4 (gray)—DSBs and structural comet.

The TT-comet assay has the potential to define four main sperm comets types, which contain different DNA damage at the origin. Figure 6A shows the four main TT-comets as observed in the human sperm cell; (1) TT-comet with tails showing a structural comet in the Y axis (Figure 6A; yellow comet); (2) TT-comet with ALSs and SSBs with long tails in Y axis (Figure 6A; pink comet); (3) TT-comet with both DSBs and SSBs-ALSs tails in the X and Y axis respectively Figure 6A; blue comet) and (4) TT-comet with DSBs showing comet tails migrating along the X axis and a structural comet in the Y axis (Figure 6B). It is interesting to highlight that the morphology of the DNA fragments on both axes (X-Neutral and Y-Alkaline) have a differently texturized chromatin (Bedford and Calvin, 1974a; Yanagimachi, 1994; Vilfan et al., 2004; Gosálvez et al., 2011).

The tail on the X axis, especially the DNA localized at the closer regions to the core, are visualized as discrete and sharp fluorescent dots, which may be representing typical DNA fragments formed from double-strand breaks at the origin; as indicated in Figure 1, these are especially evident in single neutral comets (Figures 1C,E). The comet tails of the Y axis, which represent entangled single strand DNA motifs, are more compact and the whole tail is “fuzzy” in appearance (Figures 1B,D,F).

**Figure 7:**
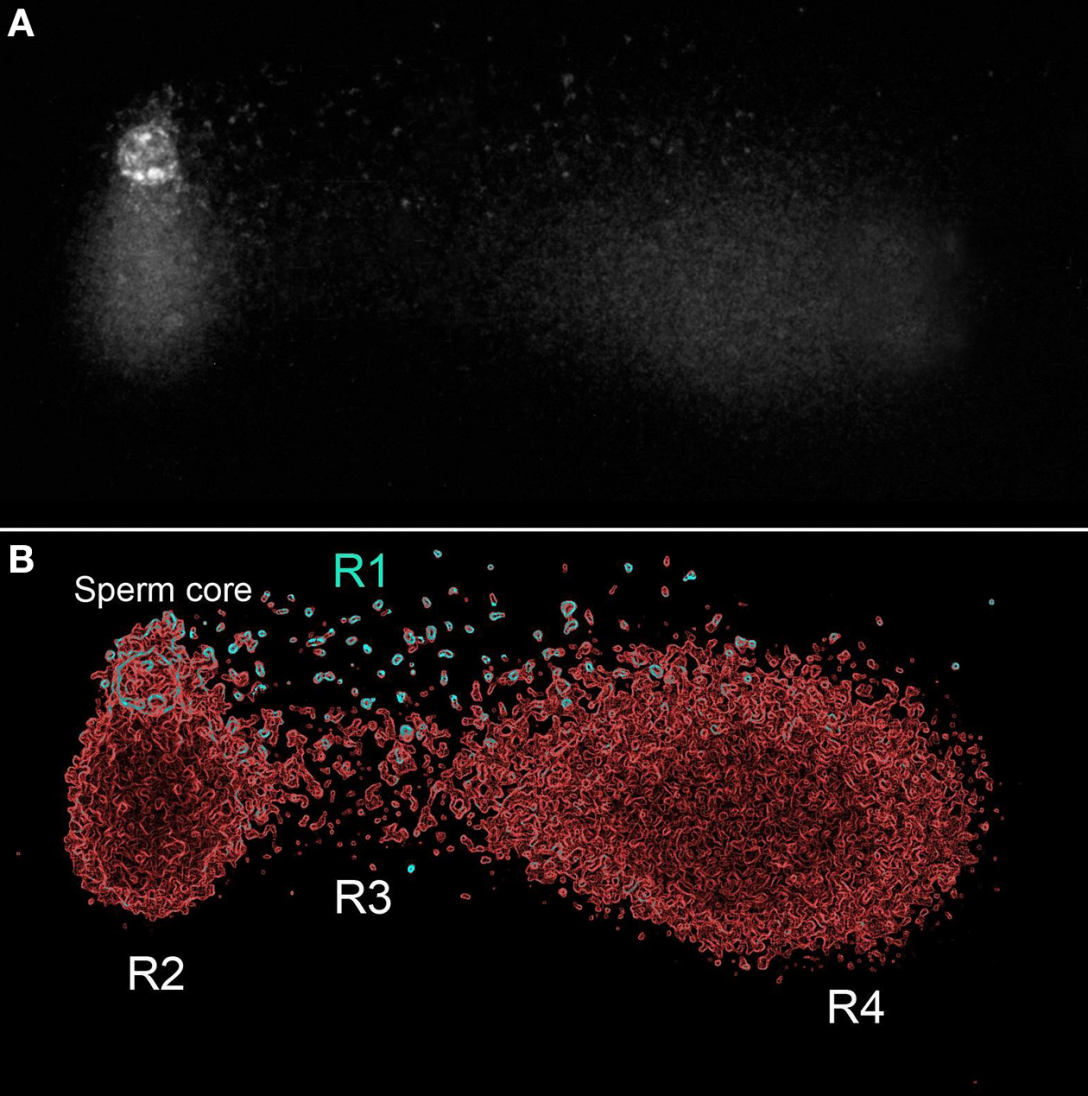
**TT comet produced after controlled double strand DNA cleavage using the restriction enzyme Alu-I (AG_CT)**. Original image **(A)**, and panel electronically enhanced **(B)**. R: different regions identified at the TT-comet. See text for detailed explanation.

The tail in the X-axis reflects the DNA fragments (DSBs) mobilized from the core that were denatured after the second electrophoretic run. The tail in the Y axis reflects comet structural in the spermatozoa (Figure 1A). To explain the possible distribution of the actual state of the DNA in this comet, we have regionalized the original image in Figure 7A in four regions (R1–R4); the results are presented on an electronically enhanced image (Figure 7B). R1 includes long DNA fragments (DSBs). DNA fragments of equivalent characteristics but shorter were mobilized under neutral conditions to the end of the comet tail. R2 includes single stranded DNA stretches originating from the constitutive comet at the original spermatozoa. R3 includes short single strand DNA stretches, which are a consequence of the DNA denaturation produced on DNA fragments displaced with the neutral comet and originally positioned at equivalent areas of R1. R4 includes single stranded DNA stretches, which are a consequence of DNA denaturation produced from enzymatic DSBs at the origin and displaced with the neutral comet. Double stranded DNA fragments at R1 located at the end of the comet tail (Figure 7B) do not exist, because they were formed by short double stranded DNA fragments that moved with neutral electrophoresis but which, subsequently, were denatured with the second alkaline electrophoresis.

## Sperm DNA damage and the detection of SSBs or DSBs for diagnostic value

High integrity of sperm DNA is an obvious requisite for normal embryonic development and a successful pregnancy. Extensive research in human and different animal species indicates that the fraction of sperm cells containing damaged DNA appears to be higher in infertile males than in fertile controls (Castilla et al., 2010). Moreover, males with poor semen quality are more likely to show a higher percentage of sperm with nuclear DNA damage than males with normal semen parameters (Gosálvez et al., 2013). Sperm DNA fragmentation can influence fertilization, embryo quality and development, blastocyst formation, and pregnancy rate; it also may lead to congenital malformations and genetic illnesses, as well as potentially increase the risk of certain cancers in related offspring (Fernández-González et al., 2008). The presence of sperm DNA damage is thought to be linked to three possible mechanisms. The first of these involves abortive apoptosis during meiosis I resulting in ejaculated spermatozoa, which, albeit defective, escape the apoptotic pathway (Sakkas et al., 2002). The second primary mechanism is defective chromatin condensation during spermiogenesis that involves inappropriate protamination and insufficient chromatin packaging. In fact, DNA breaks are produced to eliminate DNA torsional stress when substituting histones by protamines (Marcon and Boissonneault, 2004), so that unrepaired DNA breaks could persist in mature sperm. The third mechanism includes oxidative stress resulting from an imbalance between reactive oxygen species (ROS) production and antioxidant capacity (Agarwal et al., 2003).

The causes of sperm DNA damage resulting in SSBs or DSBs are extremely varied and include exposure to adverse environmental factors such as pesticides, radiation, smoking or pathological situations such as cancer, varicocele and infection. This and presumably other causes lead to the generation of sperm DNA breakage are mediated through one or a combination of the mechanisms identified above. Given knowledge as to the origin of DNA fragmentation in spermatozoa, we might also expect to see different types of DNA lesion that could possibly be predictive or diagnostic in nature. For example, nucleases, either endogenous or exogenous, should produce SSBs and/or DSBs whereas DNA breaks produced by chromatin remodeling during spermiogenesis appear to correspond to DSBs produced by topoisomerase II (Laberge and Boissonneault, 2005). Finally, ROS and other radical molecules like those derived from nitric oxide should generate mainly SSBs and many different types of DNA base damage, including mutagenic ALSs (Reiter, 2006).

The implication of sperm DNA damage in fertilization and embryo development should depend on the balance between the DNA damage from the sperm and the oocyte's repair capacity. Moreover, the type and/or complexity of DNA lesions in the different sperm can vary and this also must influence the embryonic development. After penetration into the oocyte, sperm with extensive DSBs associated with apoptotic-like processes would exceed the repair capacity of the oocyte, leading to delayed paternal DNA replication, paternal DNA degradation and arrest of embryo development (Gawecka et al., 2013). Conversely, when sperm DNA damage is composed mainly of a low level of DSBs, SSBs, abasic sites, and/or base damages, the oocyte's various specific DNA repair pathways are likely to be effective, so that the DNA of male pronucleus should function normally during syngamy and early embryonic development. Nevertheless, some mis-repaired or unrepaired DNA lesions could still potentially lead to mutations or chromosome aberrations. Unrepaired SSBs or other lesions types may also result in DSBs when DNA is replicating, leading to structural chromosomal abnormalities (Marchetti et al., 2007). If these aberrations are unstable, they are likely to affect the correct mitotic segregation of chromosomes, resulting in genomic instability and cell death, and thereby adversely affect embryo development. When DNA repair is complete, the morula and blastocyst stages can be achieved; the paternal genome should be expressed normally at this stage, so a pregnancy would be more likely. If the repair processes are not totally efficient, blastocyst arrest or spontaneous abortion may result (Fatehi et al., 2006). The differentiation of the types and levels of DNA damage that may coexist in the different sperm, therefore, provide relevant information to the study of male infertility, a technique like TT-comet could be of great value for this purpose.

## Conclusions

- When preparing for the application of TT-comet assay it is important to recognize that protamine composition in mammals is species-specific and protein lysis needs to be accordingly validated and adjusted on a species-specific basis.
- The TT-comet assay is an excellent method of discriminating between the presence of single and/or double strand breaks in the DNA in the same sperm cell.
- Structural sperm comets are correlated with the regional presence of alkali labile sites (ALS) which can be mapped using DNA breakage detection coupled with fluorescence *in situ* hybridization (DBD-FISH).
- Structural comets in the normal sperm, as revealed under alkaline DNA denaturing conditions, are a constitutive and transient circumstance, linked to the specific need for efficient chromatin packing. They are present in the sperm of all mammalian species so far analyzed.

### Conflict of interest statement

The authors declare that the research was conducted in the absence of any commercial or financial relationships that could be construed as a potential conflict of interest.

## References

[B1] Agarwal A. Saleh R. A. Bedaiwy M. A. (2003). Role of reactive oxygen species in the pathophysiology of human reproduction. Fertil. Steril. 79, 829–843. 10.1016/S0015-0282(02)04948-8 12749418

[B2] Ahnstrom G. (1988). Techniques to measure DNA strand breaks in cells: a review. Int. J. Radiat. Biol. 54, 695–707. 10.1080/09553008814552151 2902165

[B3] Allen M. L. Bradbury E. M. Balhorn R. (1997). AFM analysis of DNA protamine complexes bound to mica. Nucl. Acids Res. 25, 2221–2226. 10.1093/nar/25.11.2221 9153324 PMC146714

[B4] Collins A. R. (2004). The comet assay for DNA damage and repair: principles, applications, and limitations. Mol. Biotechnol. 26, 249–261. 10.1385/MB:26:3:249 15004294

[B5] Balhorn R. (2007). The protamine family of sperm nuclear proteins. Genome Biol. 8:227. 10.1186/gb-2007-8-9-227 17903313 PMC2375014

[B6] Bedford J. M. Calvin H. I. (1974a). The occurrence and possible functional significance of -S-S- crosslinks in sperm heads, with particular reference to eutherian mammals. J. Exp. Zool. 188, 137–156. 10.1002/jez.1401880203 4207651

[B7] Bedford J. M. Calvin H. I. (1974b). Changes in -S-S- linked structures of the sperm tail during epididymal maturation, with comparative observations in sub-mammalian species. J. Exp. Zool. 187, 181–204. 10.1002/jez.1401870202 4205051

[B8] Biegeleisen K. (2006). The probable structure of the protamine-DNA complex. J. Theor. Biol. 241, 533–540. 10.1016/j.jtbi.2005.12.015 16442565

[B9] Brooks J. E. (1987). Properties and uses of restriction endonucleases. Meth. Enzymol. 152, 113–129. 10.1016/0076-6879(87)52014-6 2821353

[B10] Castilla J. A. Zamora S. Gonzalvo M. C. Luna del Castillo J. D. Roldan-Nofuentes J. A. Clavero A.. (2010). Sperm chromatin structure assay and classical semen parameters: systematic review. Reprod. Biomed. Online 20, 114–124. 10.1016/j.rbmo.2009.10.024 20158996

[B11] Cook P. R. Brazell I. A. Jost E. (1976). Characterization of nuclear structures containing superhelical DNA. J. Cell Sci. 22, 303–324. 1002771 10.1242/jcs.22.2.303

[B12] Cortés-Gutiérrez E. I. Dávila-Rodríguez M. I. Cerda-Flores R. M. Fernández J. L. López-Fernández C. Aragón Tovar A. R.. (2014a). Localisation and quantification of alkali-labile sites in human spermatozoa by DNA breakage detection-fluorescence *in situ* hybridization. Andrologia. [Epub ahead of print]. 10.1111/and.12250 24576285

[B13] Cortés-Gutiérrez E. I. Dávila-Rodriguez M. I. Fernández J. L. Gosálvez J. Johnston S. D. López-Fernández C. (2009). Mapping alkali-labile sites in mammalian spermatozoa, in Animal Reproduction: New Research Developments, ed Dahnof Lucas T. (New York, NY: Nova Publishers), 219–231.

[B14] Cortés-Gutiérrez E. I. Dávila-Rodríguez M. I. López-Fernández C. Fernández J. L. Crespo F. Gosálvez J. (2014b). Localization of alkali-labile sites in donkey (*Equus asinus*) and stallion (Equus caballus) spermatozoa. Theriogenology 81, 321–325. 10.1016/j.theriogenology.2013.10.001 24182740

[B15] Cortés-Gutiérrez E. I. Dávila-Rodríguez M. I. López-Fernández C. Fernández J. L. Gosálvez J. (2008). Alkali-labile sites in sperm cells from Sus and Ovis species. Int. J. Androl. 31, 354–363. 10.1111/j.1365-2605.2007.00781.x 17651406

[B16] Enciso M. Iglesias M. Galán I. Sarasa J. Gosálvez A. Gosálvez J. (2011b). The ability of sperm selection techniques to remove single- or double-strand DNA damage. Asian J. Androl. 13, 764–768. 10.1038/aja.2011.46 21725332 PMC3739591

[B17] Enciso M. Johnston S. D. Gosálvez J. (2011a). Differential resistance of mammalian sperm chromatin to oxidative stress as assessed by a two-tailed comet assay. Reprod. Fertil. Dev. 23, 63. 10.1071/RD10269 21635811

[B18] Enciso M. Sarasa J. Agarwal A. Fernández J. L. Gosálvez J. (2009). A two-tailed Comet assay for assessing DNA damage in spermatozoa. Reprod. Biomed. Online 18, 609–616. 10.1016/S1472-6483(10)60003-X 19549437

[B21] Fatehi A. N. Bevers M. M. Schoevers E. Roelen B. A. Colenbrander B. Gadella B. M. (2006). DNA damage in bovine sperm does not block fertilization and early embryonic development but induces apoptosis after the first cleavages. J. Androl. 27, 176–188. 10.2164/jandrol.04152 16304212

[B22] Fernández J. L. Vázquez-Gundín F. Delgado A. Goyanes V. J. Ramiro-Díaz J. de la Torre J.. (2000). DNA breakage detection-FISH (DBD-FISH) in human spermatozoa: technical variants evidence different structural features. Mutat. Res. 45, 77–82. 10.1016/S0027-5107(00)00079-8 11006414

[B23] Fernández J. L. Vázquez-Gundín F. Rivero M. T. Goyanes V. Gosálvez J. (2001). Evidence of abundant constitutive alkali-labile sites in human 5 bp classical satellite DNA loci by DBD-FISH. Mutat. Res. 473, 163–168. 10.1016/S0027-5107(00)00146-9 11166034

[B24] Fernández J. L. Gosálvez J. (2002). Application of FISH to detect DNA damage. DNA breakage detection-FISH (DBD-FISH). Methods Mol. Biol. 203, 203–216. 10.1385/1-59259-179-5:203 12073443

[B25] Fernández-González R. Moreira P. N. Pérez-Crespo M. Sánchez-Martín M. Ramírez M. A. Pericuesta E.. (2008). Long-term effects of mouse intracytoplasmic sperm injection with DNA-fragmented sperm on health and behavior of adult offs pring. Biol. Reprod. 78, 761–772. 10.1095/biolreprod.107.065623 18199884

[B26] Gawecka J. E. Marh J. Ortega M. Yamauchi Y. Ward M. A. Ward W. S. (2013). Mouse zygotes respond to severe sperm DNA damage by delaying paternal DNA replication and embryonic Development. PLoS ONE 8:e56385. 10.1371/journal.pone.0056385 23431372 PMC3576397

[B27] Gosálvez J. García-Ochoa C. Ruíz-Jorro M. Martínez-Moya M. Sánchez-Martín P. Caballero P. (2013). At what speed does sperm deoxyribonucleic acid “die” after donor semen samples are thawed? Rev. Int. Androl. 11, 85–93 10.1016/j.androl.2013.02.005

[B28] Gosálvez J. López-Fernández C. Fernández J. L. Gouraud A. Holt W. V. (2011). Relationships between the dynamics of iatrogenic DNA damage and genomic design in mammalian spermatozoa from eleven species. Mol. Reprod. Dev. 78, 951–961. 10.1002/mrd.21394 21919111

[B29] Gosálvez J. Rodríguez–Predreira M. Mosquera A. López-Fernández C. Esteves S. C. Agarwal A.. (2014). Characterisation of a subpopulation of sperm with massive nuclear damage, as recognised with the sperm chromatin dispersion test. Andrología 46, 602–609. 10.1111/and.12118 23710631

[B30] Ichikawaa J. Tsuchimotoa D. Okaa S. Ohnoa M. Furuichib M. Sakumia K. (2008). Oxidation of mitochondrial deoxynucleotide pools by exposure to sodium nitroprusside induces cell death. DNA Repair 7, 418–430. 10.1016/j.dnarep.2007.11.007 18155646

[B30a] Johnston S. D. López-Fernández C. Gosálbez A. Holt W. V. Gosálvez J. (2009). Directional mapping of DNA nicking in ejaculated and cauda epididymidal spermatozoa of the short-beaked echidna Tachyglossus aculeatus: Monotremata. Reprod. Fertil. Dev. 21, 1008–1014. 10.1071/RD09079 19874725

[B31] Laberge R. M. Boissonneault G. (2005). On the nature of DNA strand breaks in elongating spermatids. Biol. Reprod. 73, 289–296. 10.1095/biolreprod.104.036939 15772260

[B32] Lin W. Wei X. Xue H. Kelimu M. Tao R. Song Y.. (2000). Study on DNA strand breaks induced by sodium nitroprusside, a nitric oxide donor, *in vivo* and *in vitro*. Mutat. Res. 466, 187–195. 10.1016/S1383-5718(00)00018-8 10727906

[B33] Marchetti F. Essers J. Kanaar R. Wyrobek K. J. (2007). Disruption of DNA repair increases sperm derived chromosomal aberrations. Proc. Natl. Acad. Sci. U.S.A. 104, 17725–17729. 10.1073/pnas.0705257104 17978187 PMC2077046

[B34] Marcon L. Boissonneault G. (2004). Transient DNA strand breaks during mouse and human spermiogenesis: new insights in stage specificity and link to chromatin remodelling. Biol. Reprod. 70, 910–918. 10.1095/biolreprod.103.022541 14645105

[B35] McKelvey-Martin V. J. Green M. H. L. Schmezer P. Pool-Zobel B. L. De Me'o M. P. Collins A. (1993). The single cell gel electrophoresis assay (comet assay): a European review. Mutat. Res. 288, 46–63. 10.1016/0027-5107(93)90207-V 7686265

[B36] Mezzanotte R. Ferrucci L. Vanni R. Bianchi U. (1983). Selective digestion of human metaphase chromosomes by Alu I restriction endonuclease. J. Histochem. Cytochem. 32, 553–556. 10.1177/31.4.6298309 6298309

[B37] Miller D. A. Choi J. C. Miller J. O. (1983). Chromosome localization of highly repetitive human DNAs and amplified ribosomal DNA with restriction enzymes. Science 219, 395–397. 10.1126/science.6294832 6294832

[B38] Muriel L. Segrelles E. Goyanes V. Gosálvez J. Fernández J. L. (2004). Structure of human sperm DNA and background damage, analysed by *in situ* enzymatic treatment and digital image analysis. Mol. Hum. Reprod. 10, 203–209. 10.1093/molehr/gah029 14981148

[B39] Olive P. L. Banáth J. P. (2007). The comet assay: a method to measure DNA damage in individual cells. Nat. Protoc. 1, 23–29. 10.1038/nprot.2006.5 17406208

[B40] Olivie P. L. (2006). The comet assay: an oveviw of techniques, in Methods in Molecular Biology Vol. 203, *In Situ Detection of DNA Damage: Methods and Protocols*, eds Didenki V. V. (Totowa, NJ: Humana Press Inc), 179–194.

[B41] Ostling O. Johanson K. J. (1984). Microelectrophoretic study of radiation-induced DNA damages in individual mammalian cells. Biochem. Biophys. Res. Commun. 30, 291–298. 10.1016/0006-291X(84)90411-X 6477583

[B42] Portas T. S. Johnston R. H. Arroyo F. López-Fernadez C. Bryant B. Hildebrandt T.. (2009). Frozen-thawed rhinoceros sperm exhibit DNA damage shortly after thawing when assessed by the sperm chromatin dispersion assay. Theriogenology 72, 711–720. 10.1016/j.theriogenology.2009.05.008 19560805

[B43] Reiter T. A. (2006). NO^*^ chemistry: a diversity of targets in the cell. Redox Rep. 11, 194–206. 10.1179/135100006X116718 17132268

[B44] Rivero M. T. Mosquera A. Goyanes V. Slijepcevic P. Fernández J. L. (2004). Differences in repair profiles of interstitial telomeric sites between normal and DNA double-strand break repair deficient Chinese hamster cells. Exp. Cell Res. 295, 161–172. 10.1016/j.yexcr.2003.12.031 15051499

[B45] Rivero M. T. Vázquez-Gundín F. Goyanes V. Campos A. Blasco M. Gosálvez J.. (2001). High frequency of constitutive alkali-labile sites in mouse major satellite DNA, detected by DNA breakage detection-fluorescence *in situ* hybridization. Mutat. Res. 483, 43–50. 10.1016/S0027-5107(01)00218-4 11600131

[B46] Sakkas D. Moffatt O. Manicardi G. C. Mariethoz E. Tarozzi N. Bizzaro D. (2002). Nature of DNA damage in ejaculated human spermatozoa and the possible involvement of apoptosis. Biol. Reprod. 66, 1061–1067. 10.1095/biolreprod66.4.1061 11906926

[B47] Singh N. P. McCoy M. T. Tice R. R. Schneider E. L. (1988). A simple technique for quantitation of low levels of DNA damage in individual cells. Exp. Cell Res. 175, 184–191. 10.1016/0014-4827(88)90265-0 3345800

[B48] Singh N. P. Stephens R. E. (1998). X-ray induced DNA double-strand breaks in human sperm. Mutagenesis 13, 75–79. 10.1093/mutage/13.1.75 9491398

[B50] Vilfan I. D. Conwell C. C. Hud N. V. (2004). Formation of native-like mammalian sperm cell chromatin with folded bull protamine. J. Biol. Chem. 279, 20088–20095. 10.1074/jbc.M312777200 14990583

[B51] Yamamoto N. (1969). Damage, repair, and recombination. II. Effect of hydrogen peroxide on the bacteriophage genome. Virology 38, 457–463. 10.1016/0042-6822(69)90158-5 4895144

[B52] Yanagimachi R. (1994). Mammalian fertilization, in The Physiology of Reproduction 2nd Edn., eds Knobil E. Neill J. (New York, NY: Raven Press), 189–317.

[B53] Zee Y. P. López-Fernández C. Arroyo F. Johnston S. D. Holt W. V. Gosálvez J. (2009). Evidence that single-stranded DNA breaks are a normal feature of koala sperm chromatin, while double-stranded DNA breaks are indicative of DNA damage Reproduction 138, 267–278. 10.1530/REP-09-0021 19494045

